# Nerveless and gutsy: intestinal nutrient sensing from invertebrates to humans

**DOI:** 10.1016/j.semcdb.2012.01.002

**Published:** 2012-08

**Authors:** Irene Miguel-Aliaga

**Affiliations:** Department of Zoology, Downing Street, Cambridge CB2 3EJ, United Kingdom

**Keywords:** Nutrition, Gut, Receptor, Transporter, *Drosophila elegans*

## Abstract

The increasingly recognized role of gastrointestinal signals in the regulation of food intake, insulin production and peripheral nutrient storage has prompted a surge of interest in studying how the gastrointestinal tract senses and responds to nutritional information. Identification of metabolically important intestinal nutrient sensors could provide potential new drug targets for the treatment of diabetes, obesity and gastrointestinal disorders. From a more fundamental perspective, the study of intestinal chemosensation is revealing novel, non-neuronal modes of communication involving differentiated epithelial cells. It is also identifying signalling mechanisms downstream of not only canonical receptors but also nutrient transporters, thereby supporting a chemosensory role for “transceptors” in the intestine. This review describes known and proposed mechanisms of intestinal carbohydrate, protein and lipid sensing, best characterized in mammalian systems. It also highlights the potential of invertebrate model systems such as *C. elegans* and *Drosophila melanogaster* by summarizing known examples of molecular evolutionary conservation. Recently developed genetic tools in *Drosophila*, an emerging model system for the study of physiology and metabolism, allow the temporal, spatial and high-throughput manipulation of putative intestinal sensors. Hence, fruit flies may prove particularly suited to the study of the link between intestinal nutrient sensing and metabolic homeostasis.

## Highlights

► Nutrient sensing in the gastrointestinal tract is metabolically significant. ► It is carried out by both absorptive and secretory intestinal epithelial cells. ► Gut nutrient sensors include G protein-coupled receptors and nutrient transporters. ► Nutrient sensors are evolutionarily conserved. ► Their study in *C. elegans* and *Drosophila* may shed light on their functions.

## 1 Introduction

An active role for the gastrointestinal tract in sensing and transducing nutritional information was suggested almost fifty years ago, following the observation that glucose was much more effective in raising plasma insulin concentration when supplied orally than when administered intravenously (the so-called incretin effect [1]). Subsequent studies uncovered a broader array of both local and systemic effects of luminal nutrients on, for example, gastric emptying and food intake (for a historical perspective, see [2,3]). They also established an important role for afferent nerves in mediating these responses. However, the finding that these nerves terminate in close contact with the basal lamina and do not reach the epithelial surface, thus precluding their direct activation by luminal nutrients [4], also pointed to the existence of signals from epithelial cells. Rapid progress in the past decade has resulted in the identification of intestinal epithelial receptors and transporters mediating some such signals, and has confirmed that nutrient sensing does not exclusively take place in the tongue. However, genetic loss-of-function studies have also yielded some surprising and controversial results, and the metabolic significance of some of these modes of chemosensation remains to be established.

## 2 When is a sensor not a sensor? Nutrient receptors and “transceptors”

**Fig. 1:**
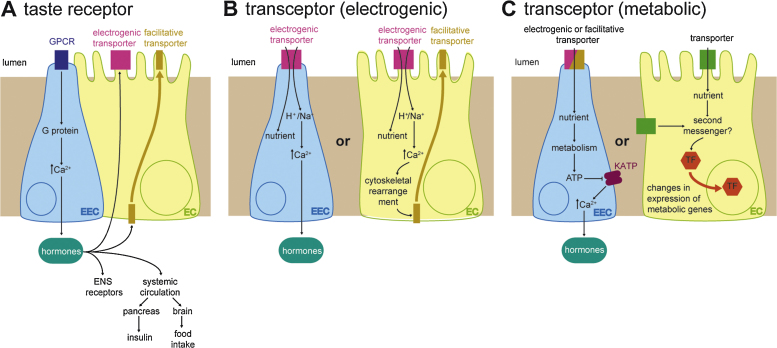
Three different modalities of intestinal nutrient sensing. (A) A taste receptor signals in EECs to affect incretin release, which, in turn, affects the expression or membrane availability of transporters in ECs. (B) Possible modes of nutrient-coupled electrogenic transport in EECs or ECs. (C) Possible mechanisms by which nutrient binding to metabolic transceptors leads to incretin release (EECs) or changes in gene expression in ECs. See text for details.

Current efforts aimed at identifying intestinal nutrient sensors are largely focused on enteroendocrine cells (EECs): specialized endocrine cells which constitute ca. 1% of the intestinal epithelial population. EECs produce peptide hormones (or incretins) which can affect neighbouring enterocytes (ECs) and nerve afferents [5]. Incretins can also be secreted systemically to affect remote functions such as pancreatic insulin production or the activity of the brain centers regulating food intake [5]. In this context, an intestinal nutrient sensor is best regarded intuitively as a receptor (typically a G protein-coupled receptor) located on the luminal side of the EEC membrane which, upon binding to a specific nutrient, triggers a signalling cascade which ultimately leads to the release of an EEC hormone (Fig. 1A and see next section for examples). This mechanism, analogous to the mode of action of taste receptors in the tongue epithelium, makes a very clear distinction between nutrient sensors (the intestinal taste receptor), the nutrient effectors (the peptide hormone) and their targets of action (for example, the expression of a nutrient transporter or its availability at the EC brush border). However, gastrointestinal chemosensation may involve additional mechanisms. Indeed, it is becoming increasingly recognized that transporters are not passive targets of nutrient signalling and can act as “transceptors”: transporters/receptors able to signal in response to nutrients to affect EEC hormone release (see next two sections for examples, and EECs in Fig. 1B and C). Signalling may be effected by the transceptor (Fig. 1C), or may result from the ion currents generated by its electrogenic transport of nutrients (Fig. 1B). Similarly, EEC hormones need not be the only effectors of nutrient sensing and there may be mechanisms of nutrient sensing that are entirely confined to ECs, carried out by transporters effecting changes in metabolic gene expression or the subcellular localization of other transporters (EC in Fig. 1B and C, for examples see Sections 3 and 5). This review aims to illustrate this broader view by providing examples of the different mechanisms by which nutritional information can be sensed and conveyed in the mammalian gastrointestinal tract, thereby inviting the reader to reflect upon the distinction between nutrient sensors, transducers and effectors of nutritional information. The review also discusses the comparative data currently available from invertebrate systems, provides examples of peptide and amino acid sensing in *Caenorhabditis elegan*s and *Drosophila melanogaster*, and highlights the potential of these model systems as future discovery tools. For recent and more exhaustive reviews of the molecular nature, pharmacology and pathophysiology of the different families of mammalian transporters and receptors I refer the reader to [3,5–10].

## 3 Intestinal sugar sensing

Complex dietary carbohydrates are broken down first by a battery of salivary and pancreatic amylases, and then by brush border enzymes which turn them into glucose, galactose and fructose [5]. Glucose is absorbed into the EC by an active transport system at the apical membrane: the Na^+^-glucose co-transporter SGLT1, which drives glucose absorption by a gradient of Na^+^ maintained by the basolateral Na^+^/K^+^-ATPase [5]. Intracellular glucose is then assumed to diffuse across the basolateral membrane via the facilitative transporter GLUT2 [5]. However, at high glucose concentrations, GLUT2 can be transiently inserted apically [11,12]. The relative contribution of the SGLT1 and GLUT2 transport systems in these circumstances will need to be clarified [12], although the recently published phenotypes of SGLT1-deficient mice (as severe as the effects of SGLT1 mutation in humans [13,14]) suggest that SGLT1 is the primary pathway for the transport of glucose across the brush border membrane during glucose mass absorption [15].

### 3.1 A taste of honey: the role of sweet taste receptors

The role of glucose sensing on EEC hormone release has been the main focus of attention for the past couple of decades since it became apparent that the surplus of insulin resulting from the incretin effect was due to the combined effects of two such hormones (glucagon-like peptide-1 (GLP-1) and gastric inhibitory peptide (GIP) [16]). A “classic” nutrient-sensing mechanism involving sweet taste receptors like those found in our tongue has been described, following the finding of alpha-gustducin (a G protein alpha-subunit involved in taste transduction in lingual taste cells [17,18]) in the EECs of rat, mouse and human [19–23], where it is co-expressed with sweet taste receptors [21,22]. An intestinal role for these sweet taste receptors was suggested by the finding that antagonists of sweet-taste receptors block GLP-1 and GIP secretions of human and mouse endocrine cell lines [21,22], and was further supported by mice lacking alpha-gustducin or the sweet taste receptor T1R3, which were found neither to increase GLP-1 or GIP secretion nor to upregulate SGLT1 in response to dietary sugar or sweeteners [21,22]. However, a role for the intestinal (as opposed to lingual) taste signalling components in EEC hormone release has only been directly demonstrated for alpha-gustducin, by showing reduced GLP-1 secretion following glucose infusions into the stomach lumen or isolated duodenum of the alpha-gustducin mouse mutants [21]. On the basis of these observations, the model illustrated in Fig. 1A has emerged, whereby luminal glucose activates the sweet taste receptors T1R2 and T1R3 and its downstream G protein gustducin in EECs, leading to release of GLP-1 and GIP – presumably through the action of second messengers like those in lingual taste receptor cells, which affect cAMP and intracellular Ca^2+^ levels and lead to depolarization [24]. Amongst a broad range of systemic effects and local actions on both ECs and nerve afferents [10,16,25] these EEC hormones would affect glucose absorption by upregulating SGLT1 expression and promoting apical GLUT2 insertion in ECs. Consistent with this idea, a high-sugar diet and sweeteners have been shown to upregulate SGLT1 in a gustducin and T1R3-dependent manner [22], and gut-expressed taste receptors and hormones have also been implicated in the sugar-induced GLUT2 trafficking from the basolateral to the brush border membrane [26,27]. However, this latter action of taste receptors on EC transporters has also been proposed to occur in a cell-autonomous fashion without an involvement of EEC hormones, given that some (but not all [21]) groups have reported expression of taste signalling components in ECs or EC cell lines ([27,28] and see [12,25] for two different views). The relative contribution of the two mechanisms might differ in specific portions of the digestive tract (e.g. duodenum vs. jejunum), given that the expression of these taste signalling molecules shows regional differences (see [12] for a review).

### 3.2 SGLT1 and GLUT2: sugar sensing by electrogenic or metabolic transceptors

The relatively mild phenotypes of mice lacking taste signalling components [21,22], together with the finding that artificial sweeteners do not induce GLP-1 secretion in humans [3,29–31], suggest the existence of additional sugar-sensing mechanisms. Recent studies suggest that sugar transporters are not only the targets of intestinal taste receptor signalling, but may also play active roles in nutrient sensing by acting either as electrogenic or metabolic transceptors (Fig. 1B and C, respectively) to control EEC hormone release, expression of other transporters or metabolic gene expression. Firstly, SGLT1 (and, possibly, GLUT2 [32]) may act in EECs to regulate hormone release. Consistent with this idea, SGLT1 has been detected in GIP and GLP-1-producing EECs [15,33,34] and EEC hormone secretion is reduced in mice lacking SGLT1 [15]. How glucose binding to SGLT1 leads to EEC hormone release is an area of active investigation. The electrogenic symport transport of Na^+^ may couple glucose entry to GLP-1 secretion through membrane depolarisation, electrical activity and Ca^2+^ entry [34–36]. In this case, SGLT1 would act as an electrogenic transceptor in EECs (Fig. 1B, EEC). Alternatively (or in parallel), depolarization and Ca^2+^ influx may result from the closure of metabolism-dependent KATP channels [34], analogous to those regulating insulin secretion in pancreatic beta-cells in response to glucose metabolism [37] (EEC in Fig. 1C). A second possible site of action for SGLT1 as a nutrient sensor is the EC itself, where the SGLT1-mediated electrogenic transport of Na^+^ would depolarize the apical membrane to induce rapid influx of Ca^2+^ [38,39]. This would lead to phosphorylation of cytoskeletal components, enabling apical GLUT2 insertion and thereby allowing additional transport capacity [40] (EC in Fig. 1B).

Finally, sugar transporters have also been proposed to act as metabolic transceptors in the EC. In hepatic cells, the intracytoplasmic loop of GLUT2 can signal by binding to karyopherin alpha2, a receptor involved in nuclear import, to stimulate glucose-sensitive gene transcription [41–43] (EC in Fig. 1C). More recently, experiments with different GLUT2 constructs in an EC cell line have suggested a similar mode of action [28].

## 4 Intestinal protein sensing

Unlike complex sugars, proteins are broken down into products of diverse chemical nature, including di- and tri-peptides and a mixture of amino acids [5]. These diverse digestion products require a larger number of both apical and basolateral transport systems with different substrate specificity and ion dependency, the molecular nature of which has only recently been established [5,8]. Consequently, little is known about the nature and significance of intestinal protein sensing mechanisms other than they are likely to be important; changes in protein availability have profound effects on gene expression and metabolism, and have been shown to regulate important signalling pathways such as those involving target of rapamycin (TOR) complex 1 or the general control non-derepressing kinase 2 (GCN2) [44–48]. In the context of the gastrointestinal tract, the products of protein hydrolysis have been shown to stimulate secretion of EEC peptides [49,50].

Mechanistically, intestinal protein sensing may make use of the same three mechanisms described for sugar sensing and depicted in Fig. 1. Indeed, GPR93 has been proposed to act like a taste receptor (Fig. 1A), on the basis that its expression and activation promote cholecystokinin (CCK) secretion, at least in a CCK-secreting EEC line [51]. Secondly, both amino acid and dipeptide transporters may function as electrogenic transceptors in EECs (Fig. 1B). For example, the amino acid glutamine is a potent trigger of GLP-1 release in a murine EEC line upon binding to the Na^+^-dependent amino-acid transporter SLC38A2 (a system A Na^+^-neutral amino acid transporter, expressed basolaterally) [52], and the apical dipeptide transporter PEPT1 can induce dipeptide-triggered membrane depolarization and secretion in another EEC line [53]. Finally, amino acid transporters also have the potential to function as metabolic transceptors (Fig. 1C). In fact, the “transceptor” concept was coined after the finding that Ssy, a yeast amino acid permease, operates in conjunction with two other membrane proteins to promote the activation of two transcription factors and consequent transcription of amino acid permease genes upon binding of amino acids [54,55]. More recently, a similar transceptor role has been reported for the broad-range amino acid transporter Gap1 – in this case acting through PKA activation [56]. Although some evidence suggests that amino acid metabolic transceptors may also function in *Drosophila* (Path transporter, see Section 6) and mammals [55], their involvement in intestinal amino acid sensing has not been investigated.

Generally, and as in the case of sugar sensing, both the physiological significance and relative importance of peptide/amino acid receptors and transporters remain to be established, and their genetic manipulation is revealing some surprises. For example, even though peptide (as opposed to amino acid) absorption appears to be the most significant entry route into the EC [5] and both the expression and/or trafficking of PEPT1 are subject to complex hormonal and dietary regulation [57–59], mice lacking PEPT1 are viable and have a relatively mild phenotype [60,61]. By contrast, disruption of either apical or basolateral amino acid transporters can have more dramatic consequences, either in model systems or when mutated in humans (for reviews see [7,9]). It will be important to determine whether and how homeostatic mechanisms are deployed in these loss-of-function situations, which will require both the temporal and spatial control of gene expression (see Sections 6 and 7).

## 5 Intestinal fat sensing

Lipid intestinal absorption takes place after luminal lipases break down triacylglycerols (TAG) into fatty acids (FAs) and monoglycerides. Inside the EC, FAs are incorporated into TAG, phospholipids and cholesteryl esters, which are packaged together with apoproteins into chylomicrons to be secreted out of the EC into the circulation. The existence of intestinal lipid sensors has been postulated on the basis of the major effects of lipids on EEC hormone secretion and inhibition of gastric emptying [62], and experiments with gut fat infusions and FAs of defined lengths have pointed to an important role for long-chain FAs [63 and references therein, 64]. Since then, several candidate lipid sensors have been identified, most of which are GPCRs (mechanism as shown in EEC in Fig. 1A, for a comprehensive review see [3]). GPR120 and GPR40 are probably the two best characterized examples. GPR120 is present in human and mouse intestine, where it co-localizes with GLP-1 predominantly in colonic EECs [65]. Unsaturated long-chain FAs have been found to increase intracellular Ca^2+^ and stimulate GLP-1 in GPR-120-expressing cells. More importantly, colonic administration of alpha-linolenic acid is associated with an increase in circulating GLP-1, which is severely reduced in mice lacking GPR120 [65]. Similarly, GPR40 has been found to co-localize with GLP-1, GIP and CCK in mouse EECs [66,67] and its characterization in wild-type and GPR40 knockout mice has confirmed its contribution to the long-chain FA-evoked CCK release [67].

Non-GPCR-mediated mechanisms are much less understood. The fatty acid translocase FAT CD36 is expressed in intestinal brush borders [68] and mediates long-chain FA uptake in the small intestine [69]. A nutrient sensing role for CD36 in ECs, extrapolated from its sensing actions in the tongue epithelium [70], may involve trafficking between the plasma membrane and organelles to route and package FAs into chylomicrons [71]. The apolipoprotein A-IV in these chylomicrons appears to be essential to convey information about the contents of the intestinal lumen via the vagus to the CNS [72,73]. CD36 also seems to be essential for the production of the lipid messenger oleoylethanolamide, which suppresses food intake via activation of the nuclear receptor peroxisome-proliferator-activated receptor-α [74,75]. Thus, several novel metabolic transceptor-like mechanisms (Fig. 1C) involving CD36 have been proposed. It will be of interest to establish their metabolic significance and uncouple the nutrient-triggered effects of the receptor in taste bud cells from those in the intestine.

## 6 In search of metabolically relevant intestinal sensors: nutrient sensing in invertebrate guts

Pharmacological and genetic approaches have begun to shed light on the metabolic significance of this large number of candidate nutrient sensors. However, they have also suggested functional redundancy, and have highlighted the difficulties of characterizing the enteric subpopulations in which they function without interfering with their non-intestinal roles. Invertebrate model systems such as *C. elegans* and *D. melanogaster* allow the fast and high-throughput downregulation of any gene, either in wild-type or sensitized (e.g. diabetic, obese) genetic backgrounds. Consequently, they could provide an entry point into both identifying new sensors and dissecting the functional significance of those already known in mammals. For example, recent findings in *C. elegans* point to an additional, GLUT-independent sugar efflux route involving a new class of sugar transporters (SWEETs [76,77]). *Drosophila* would appear particularly suited to these approaches: it feeds on a complex diet and adapts its food intake and preference to its metabolic state [78–81]. It also has a regionally specialized digestive tract consisting of the same major cell types as the mammalian intestine (ECs, EECs, intestinal stem cells, visceral muscles and enteric neurons) [79,82]. Importantly, gene function can be abrogated or restored specifically in each of these intestinal cell populations with temporal resolution.

Surprisingly, the functions of invertebrate intestinal transporters and taste receptors remain largely unexplored. This is in spite of the molecular evolutionary conservation of several of the protein families involved (perhaps with the exception of the signalling machinery involved in taste transduction [83,84]). Indeed, GLUT- and SGLT1-like genes and/or transporter activities have been described in the intestine of insects [85–89]. GLUT-like genes have also been cloned (but not characterized) in both *C. elegans* and *Drosophila* (Geoff Holman, personal communication, and [90]), and gustatory receptors like those found in taste cells have recently been reported in *Drosophila* EECs [91]. Similarly, many of the peptide and amino acid transport systems (both apical and basolateral) are present in both model systems (see Table 1 and specific examples below). The following two examples illustrate how the few available studies in invertebrates are revealing functional conservation and are linking specific transporter families to important signalling pathways, thus shedding light on their mode of action.

### 6.1 The *C. elegans* orthologue of the peptide transporter PEPT1

Mutation of the *C. elegans* PEPT1 (*opt-2/pep-2/cptb*, hereby referred to as *pep-2*) provided the first i*n vivo* evidence for the metabolic significance of a peptide transporter. Like its mammalian counterpart, *pep-2* functions as an H^+^-coupled oligopeptide transporter [92,93]. Worms lacking *pep-2* grow less, are developmentally delayed and have fewer progeny [94,95]. *pep-2* expression is confined to the worm's digestive tract [95], where it is absolutely required for the intestinal uptake of dietary dipeptides, and this function cannot be compensated for by other amino acid transporters – at least with regard to growth [94]. Metabolic profiling has not only revealed defects in amino acid metabolism [96] but has also uncovered a link between dipeptide transport and lipid metabolism [97]. Contrary to earlier findings [76,95], the latter study found that *pep-2* loss of function was associated with increased total body fat, probably resulting from more efficient intestinal absorption of dietary FAs. A genetic interaction between *pep-2* and TOR pathway mutants has also been described, whereby the absence of *pep-2* increases the lifespan of insulin receptor mutants [94]. Together, these experiments point to a central role for the EC in balancing protein and lipid absorption and coupling nutritional state with important metabolic pathways, but further experiments will be needed to clarify the link between these processes and identify whether the relevant sensor is *pep-2* itself or an additional transporter/receptor.

In light of these phenotypes and the important roles for mammalian PEPT1 suggested by pharmacological and dietary experiments (see Section 4), the recent finding that mice lacking PEPT1 are relatively healthy was very surprising [60,61]. Differences in the diet and amino acid transporter repertoire between worms and mice may account for the different phenotypes: a hypothesis that could be tested in flies, where a PEPT1-like gene with expression in the intestinal brush border has also been described [98]. The conservation of at least some amino acid transport systems in this model system (Table 1) may help identify the homeostatic mechanisms that might mask the PEPT1 loss-of-function phenotypes in mice.

### 6.2 Amino acid transporters, TOR signalling and growth regulation in insects

In addition to apical di- and tri-peptide transporters, homologues of mammalian genes belonging to multiple families of both apical and basolateral amino acid transporters have also been reported in several insects, and most have been shown to be expressed in the intestine (Table 1 and references therein). Although data regarding their transporter activity is generally scarce, a few studies have revealed functional conservation [99–101], while others have pointed to the existence of additional insect-specific subfamilies, especially within the Na^+^ symporter superfamily SLC6 [102–106].

Genetic mutation of members of three different transporter classes in *Drosophila* has revealed a link between amino acid transport and the regulation of growth. The transporter may be required to promote growth cell autonomously, as in the case of the PAT-related transporter *pathetic* (*path*) [99]. But the growth-promoting effect can also be non cell-autonomous, as shown for the neutral amino acid exchanger *minidiscs* (*mnd*) and the cationic amino acid transporter *slimfast* (*slif*) (Table 1) [107,108]. Indeed, in developing larvae, *slif*-dependent signalling in the fat body (an organ with functional similarities to the mammalian liver and white adipose tissue [109]) modulates a humoral signal able to affect insulin signalling and growth in other tissues. The requirement for *slif* may additionally extend into adult life, as suggested by the effects of its downregulation on mosquito reproduction [110]. Interestingly, both *slif* and *path* genetically interact with the TOR signalling pathway [99,107], which has additionally been shown to modulate Slif subcellular trafficking [111], thereby coupling cell signalling events with the molecular machinery regulating amino acid availability. While these studies have provided the first *in vivo* evidence for the importance of these three transport systems, their significance in the intestine remains to be established, as does their mode of action. In this regard, a transceptor-like nutrient sensing role has been proposed for *path* on the basis of its transport properties and saturation dynamics in Xenopus oocytes [99]. These findings suggest an active nutrient sensing role and argue against a more passive effect of amino acid transport on the energetic status of the cell and, indirectly, on TOR signalling.

## 7 Conclusions and outlook

The gastrointestinal tract is becoming an excellent system for the study of nutrient sensing, which can now be used to link molecularly characterized membrane receptors and/or transporters with defined cellular changes, the secretion of signalling hormones, and their metabolic consequences. While a view is emerging of the intestine as a nutritional homeostat, the relative contribution of potential nutrient sensors will need to be clarified. To this end, it will be important to:

1) *Increase our understanding of the intestinal targets of nutritional regulation*. These may extend beyond incretin release to mechanisms entirely confined to ECs. Characterization of these EC-based mechanisms regulating nutrient absorption and metabolite trafficking will help distinguish passive effects of nutrient transport on energetic status from active signalling events.
2) *Interfere with the function of a nutrient sensor in the absence of homeostatic adaptations*. Existing pharmacological data will need to be complemented by genetic *in vivo* analyses in which the expression of the potential nutrient sensor can be manipulated with temporal resolution and specifically in the intestine.
3) *Characterize the homeostatic mechanisms that may be masking loss-of-function phenotypes*. This is essential if nutrient sensors are to become effective drug targets for the treatment of metabolic conditions, and may involve interfering with receptors/transporters in groups rather than singly.

## Figures and Tables

**Table 1:** Human intestinal peptide and amino acid transporter systems with described invertebrate homologues. Modified from [5] to include invertebrate homologues and their intestinal expression. Abbreviations: A (apical), BL (basolateral), AA (amino acid), *Dm* (*Drosophila melanogaster*), *Aa* (*Aedes aegypti*), *Ag* (*Anopheles gambiae*), *Ce* (*C. elegans*), NI (not investigated).

System	Molecular identity (genes)	Substrates	Ion dependency	Invertebrate gene	Expression in intestinal epithelium
PAT (A)	PAT1 (*SLC36A1*)	Small neutral AAs (Gly, Ala, Pro)	Electrogenic (H^+^-coupled)	*Dm path*[99]	Yes [99]
				*Dm CG1139*[99]	NI

B^0^ (A)	B^0^AT1 (*SLC6A19*)	Broad, neutral AAs (not imino or beta AAs)	Electrogenic (Na^+^-coupled)	Aa AAT1 [102] ^*^	Yes [102]
				Dm NAAT1 [105,106] ^*^	Yes [105,106]
				Ag NAT6 and AgNAT8 [112–114] ^*^	Yes [112–114]
L (BL)	LAT2 (*SLC7A8*) and 4F2hc (aka CD98hc, *SLC3A2*)	Neutral AAs (not imino)	None (neutral AA exchange)	*Dm CG2791* (*CD89hc*-like) [101]	NI
				*Dm mnd*[108]	Yes [108]
				*Dm CG1607, gb, JhI-21* and *CG9413* (LAT1/LAT2-like) [101]	NI

y+ (BL)	CAT1 (*SLC7A1*)	Cationic AAs (Lys, Arg, ornithine)	None, electrogenic (energized by negative membrane potential). AA import into EC	*Dm slif*[107]	NI
				*Aa slif* (aka *Aa CAT1*) [100,110]	Yes? [100,110]
				*Aa CAT2*[110]	NI
					

PEPT1 (A)	PEPT1 (*SLC15A1*)	Dipeptides, tripeptides	Electrogenic (H^+^-coupled)	*Dm yin* (*opt1*) [98]	Yes [98]
				*Ce pep-2*[92–95]	Yes [95]

^*^Although see main text and, specifically, [102,106,113] and references therein for different substrate specificity and ion dependence, and for additional B^0^-like transporters specific to insects (based on sequence and/or transport properties).
